# Decision Fusion-Based Deep Learning for Channel State Information Channel-Aware Human Action Recognition

**DOI:** 10.3390/s25041061

**Published:** 2025-02-10

**Authors:** Domonkos Varga

**Affiliations:** Nokia Bell Labs, 1082 Budapest, Hungary; domonkos.varga@nokia-bell-labs.com

**Keywords:** WiFi channel state information, human action recognition, deep learning

## Abstract

WiFi channel state information (CSI) has emerged as a promising modality for human action recognition due to its non-invasive nature and robustness in diverse environments. However, most existing methods process CSI channels collectively, potentially overlooking valuable channel-specific information. In this study, we propose a novel architecture, DF-CNN, which treats CSI channels separately and integrates their outputs using a decision fusion (DF) strategy. Extensive experiments demonstrate that DF-CNN significantly outperforms traditional approaches, achieving state-of-the-art performance. We also provide a comprehensive analysis of individual and combined CSI channel evaluations, showcasing the effectiveness of our method. This work establishes the importance of separate channel processing in CSI-based human action recognition and sets a new benchmark for the field.

## 1. Introduction

With the proliferation of wireless networks and the increasing demand for context-aware applications, WiFi channel state information (CSI) has emerged as a promising technology for human action recognition. Unlike traditional vision-based approaches that require dedicated cameras and raise privacy concerns, WiFi CSI-based systems leverage existing wireless infrastructure to capture human movements through their impact on wireless signals.

WiFi CSI provides fine-grained information about how wireless signals propagate between transmitter and receiver pairs, including amplitude and phase information across multiple subcarriers. When human activities occur within the wireless environment, they create distinctive patterns in the CSI measurements due to signal reflection, refraction, and scattering. These patterns can be analyzed to recognize and classify different human actions without requiring direct line-of-sight or specialized sensing equipment.

Recent advances in deep learning and signal processing have significantly enhanced the capability of CSI-based human action recognition systems. These systems have demonstrated success in various applications, including smart homes [1], healthcare monitoring [2,3], elderly care [4], and security surveillance [5]. The non-intrusive nature of WiFi sensing [6], combined with its ubiquitous availability and low cost, makes it an attractive alternative to traditional sensing modalities. However, CSI-based action recognition faces several challenges [7], including environmental dynamics, device heterogeneity, and the complex relationship between human movements and CSI variations. Additionally, the high dimensionality of CSI data and the need for robust feature extraction methods present significant research opportunities in this field.

### 1.1. Contributions

The primary contribution of this work is the development of a novel structure that processes CSI channels separately, as opposed to prior approaches that treated them collectively as a single input. By leveraging decision fusion (DF), the proposed method effectively combines information from individual channels, resulting in a significant performance improvement over existing techniques. This innovative approach demonstrates the advantages of treating CSI channels independently and highlights the potential of DF for robust human action recognition.

### 1.2. Structure of This Paper

Section 2 introduces the theoretical foundations of WiFi CSI, explaining the channel frequency response, subcarrier information, and the relationship between human movements and CSI variations. Section 3 presents a comprehensive review of related work in WiFi CSI-based human action recognition, including recent advances in deep learning approaches. Section 4 details our materials and methods, describing the experimental setup and the proposed recognition algorithm.

## 2. Preliminaries on WiFi CSI

WiFi channel state information (CSI) is a detailed representation of the wireless communication channel between a transmitter and a receiver. Unlike received signal strength indicator (RSSI), which provides a single scalar value for the signal power, CSI captures the amplitude and phase information of multiple subcarriers across the channel, offering a high-resolution view of the propagation environment. CSI data are extracted at the physical layer of WiFi communication and provide insight into the interaction of transmitted signals with the surrounding environment. This includes the effects of multipath propagation, interference, and obstacles, which alter the signal during transmission. CSI describes amplitude and phase information for each subcarrier in orthogonal frequency division multiplexing (OFDM) systems. For each packet transmission, CSI can be expressed as follows:

(1) $$H\left(f,t\right)=\left|H\left(f,t\right)\right|e^{j\theta (f,t)},$$

where H(f,t) is the CSI, which represents how a wireless signal is modified by the channel between transmitter and receiver. It is a complex value that captures the following:

1. How much the signal’s amplitude changes (attenuation);
2. How much the signal’s phase shifts;
3. These changes for each frequency (f) at each time point (t).

Furthermore, θ(f,t) represents the phase. In modern WiFi systems (IEEE 802.11n/ac/ax [8]), CSI provides detailed channel measurements across multiple subcarriers and antenna pairs, forming a complex matrix. For human action recognition, CSI measurements capture subtle environmental changes caused by human movements, as these movements affect signal propagation through multipath effects, reflection, and scattering. Each CSI sample typically contains 30–60 subcarriers per antenna pair, providing fine-grained information about channel characteristics that can be used to detect and classify human activities.

## 3. Related Work

As already mentioned, WiFi CSI-based human action recognition has emerged as a significant area of research, leveraging the unique characteristics of WiFi signals to detect and interpret human activities. This literature review synthesizes recent advancements in the field, highlighting the methodologies, applications, and challenges associated with using CSI for human action recognition. The fundamental premise of utilizing WiFi CSI for human activity recognition lies in its ability to capture minute variations in the wireless signal caused by human movements. The propagation of WiFi signals is influenced by both static and dynamic objects in the environment, with human activities introducing distinct patterns in the CSI data [9,10]. This capability allows for the development of systems that can monitor activities in a non-intrusive manner, preserving privacy while providing valuable data for applications in smart homes, healthcare, and security [11,12].

Recent studies have demonstrated the effectiveness of deep learning frameworks in enhancing the accuracy of human action recognition systems based on WiFi CSI. For instance, Ding and Wang [13] proposed a deep recurrent neural network (RNN) that exploits the temporal dynamics of CSI to recognize various human activities. In contrast, Yousefi et al. [14] proposed a long short-term memory (LSTM) [15] network for the classification of human actions based on CSI signals. Similarly, Yang et al. [16] introduced an LSTM, but an attention mechanism [17] was also applied by the authors. In [18], Schäfer et al. integrated an LSTM and a support vector machine (SVM) [19] into a specific structure for CSI-based human action recognition. Similarly, Zhou et al. [20] devised a hybrid system but features from the CSI signals were extracted with the help of a deep convolutional neural network (CNN) and were classified with an SVM. Huang et al. [21] also extracted features with a deep CNN but the authors classified the signals with an LSTM. Sheng et al. [22] chose a similar layout but a bi-directional LSTM was used for signal classification. A similar bi-directional LSTM-based method was published in [23]. In contrast, Ge et al. [24] combined a vision transformer [25] with an LSTM. Yang et al. [26] introduced two signal enhancement techniques, i.e., *N*-iteration signal enhancement (NISE) and *P*-signal enhancement (PSE), to improve recognition performance. Zhou et al. [27] emphasized the potential of transforming raw CSI signals into image formats to facilitate fine-grained gesture recognition, leveraging CNNs for precise classification. This method capitalizes on the spatial and temporal patterns inherent in the CSI data, allowing CNNs to perform effectively in recognizing subtle differences in human gestures. Jiao and Zhang [28] transformed CSI signals into images by applying Gramian angular fields which were classified into different human actions with the help of a CNN. This approach was further developed in [29] by fine-tuning on ImageNet [30] database pre-trained CNNs. The challenges associated with data scarcity and environmental variability have also been addressed through advanced techniques. For example, Zhang et al. [31] proposed data augmentation strategies to synthesize diverse activity data, which mitigates the overfitting issues commonly faced in training deep learning models with limited datasets. Such strategies are crucial for enhancing the robustness and generalizability of CNN-based models in real-world applications.

Moreover, the literature indicates that the use of CSI extends beyond simple activity recognition to encompass gesture recognition and human identification. For example, Li et al. [32] developed a gesture recognition system that utilizes CSI extracted from smartphones, demonstrating the versatility of WiFi-based systems in recognizing specific hand movements. Additionally, Zou et al. [33] explored the application of convex tensor shapelet learning for human identification, further illustrating the diverse methodologies being employed in this field. This breadth of application underscores the adaptability of WiFi CSI in various contexts, from healthcare monitoring to interactive gaming [34]. The integration of WiFi CSI with other sensors, such as vision-based systems, has gained traction as researchers seek to enhance the robustness and accuracy of human action recognition systems. For instance, Guo et al. [35] proposed a hybrid approach that combines WiFi signals with Kinect-based skeleton data to improve activity recognition in challenging environments characterized by occlusion and varying lighting conditions. This multimodal strategy leverages the strengths of both sensing modalities, allowing for more accurate and reliable human activity detection. Similarly, Chen et al. [36] explored the use of WiFi CSI alongside skeleton data captured by a Kinect camera, employing a bidirectional gated recurrent unit model to effectively extract features from the combined data. This integration not only enhances recognition accuracy but also provides a richer context for understanding human actions.

Despite the promising advancements, challenges remain in the deployment of WiFi CSI-based recognition systems. Environmental factors, such as changes in furniture layout or the presence of multiple individuals, can significantly affect the accuracy of recognition algorithms [37]. Furthermore, the need for robust feature extraction methods is critical, as the effectiveness of recognition systems often hinges on the quality of the extracted features from the CSI data [14]. Recent works have addressed these challenges by proposing novel algorithms and frameworks that enhance the robustness of CSI-based recognition systems [38,39].

## 4. Materials and Methods

This section outlines the dataset and methodology employed in this study. First, we describe the publicly available CSI-HAR dataset used for evaluating the proposed approach, highlighting its key characteristics and suitability for human activity recognition in Section 4.1. Next, we introduce the proposed method in detail, including the architectural design, processing pipeline, and evaluation protocol, emphasizing its ability to leverage the unique properties of CSI data for accurate and efficient activity recognition in Section 4.2.

### 4.1. Materials

We utilized the publicly available CSI-HAR dataset presented in the study by Fard Moshiri et al. [40]. This dataset was collected using a Raspberry Pi 4 [41] and Nexmon CSI Tool [42] in an indoor environment and includes seven human activities: walk, run, fall, lie down, sit down, stand up, and bend. Each activity was performed 20 times by three participants of different ages, resulting in 420 samples. The dataset captures CSI data with 52 subcarriers and consists of between 600 and 1100 rows per activity, depending on the duration. The CSI data reflect signal variations caused by human movements and are suitable for evaluating methods in WiFi-based human activity recognition. Additionally, the dataset is accessible on GitHub, making it a valuable resource for reproducibility and further research in the field.

Besides CSI-HAR [40], we also utilized the Basic Knife Skills Database [43]. Moghaddam et al. [43] used an ESP32 microcontroller [44] and iPhone 12 mini to collect CSI and RSSI data from WiFi signals to detect different knife-based cooking activities, i.e., chop, French cut, cube, slice, julienne, and mince. The data were collected from two participants performing knife activities.

### 4.2. Methods

**Figure 1:**
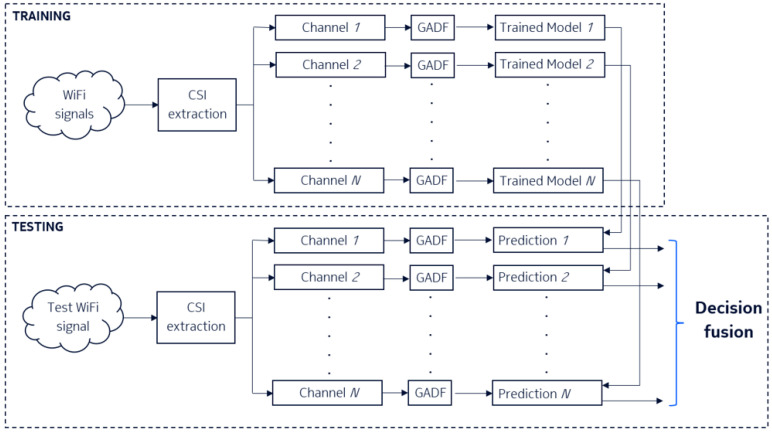
Workflow of the proposed WiFi CSI-based human action recognition system. The system is divided into two phases: training (top) and testing (bottom). In the training phase, WiFi signals are processed to extract channel state information, which is transformed into Gramian angular difference field (GADF) representations for each channel. Channel-specific models are trained independently using the GADF features. In the testing phase, the CSI from test signals undergoes the same transformation, and predictions are generated by the corresponding trained models for each channel. The final classification is obtained through a decision fusion mechanism that aggregates channel-specific predictions.

Figure 1 depicts the diagram that illustrates the workflow of the proposed method. The proposed system for human action recognition leverages WiFi channel state information and employs a channel-wise feature extraction and classification strategy, as illustrated in the workflow. The methodology is divided into two distinct phases: training and testing.

In the training phase, raw WiFi signals are collected first to capture spatiotemporal variations caused by human actions. Next, CSI is extracted from the raw WiFi signals, providing fine-grained channel-specific data. For each CSI channel (i∈{1,2,…,N}), the time-series data are transformed into a two-dimensional representation using the Gramian angular difference field (GADF). GADF is a type of Gramian angular field (GAF) and represents the angular difference between time points in the signal. GAF is a method to encode time series data into a matrix representation by transforming the data into polar coordinates and then computing the Gramian matrix [45]. The GAF representation preserves temporal dependencies and can be used as an image-like input for machine learning models. Similarly, the GADF transforms time series data into image-like representations, enabling the application of powerful computer vision techniques to time series analysis. By converting temporal patterns into spatial relationships, GADF helps detect complex patterns in various applications, from financial forecasting [46] to human activity recognition [29]. This transformation encodes temporal dependencies and preserves information about signal dynamics in a visual format suitable for deep learning. Specifically, a Gramian angular difference field (GADF) is a 2D representation of a 1D time-series signal [47]. Below is a step-by-step guide to generate a GADF [48]:

1. Normalization of the signal. The 1D signal *x* must be scaled into the range [−1, 1]. This is essential because the GADF uses the arccosine function, which requires input within this range. Formally, we can write:
  (2) $$x_{norm}=\frac{x-\min(x)}{\max(x)-min(x)}\cdot 2-1.$$
2. Transformation to polar coordinates. Each normalized value has to be converted into its angular representation (ϕ) using the arccosine function:
  (3) $$\phi =\arccos(x_{norm}).$$
  This maps the time-series data to angles in the range [0, π].
3. Creation of the angular difference matrix. Pairwise angular differences between all time points are computed as follows:
  (4) $$\Delta \phi_{ij}=\phi_{i}-\phi_{j}.$$
4. Generation of GADF. Finally, the GADF is determined as the cosine of the angular differences:
  (5) $$GADF_{ij}=\sin\left(\phi_{i}+\phi_{j}\right)\cdot sin\left(\phi_{i}-\phi_{j}\right).$$
  This results in a 2D matrix.

**Figure 2:**
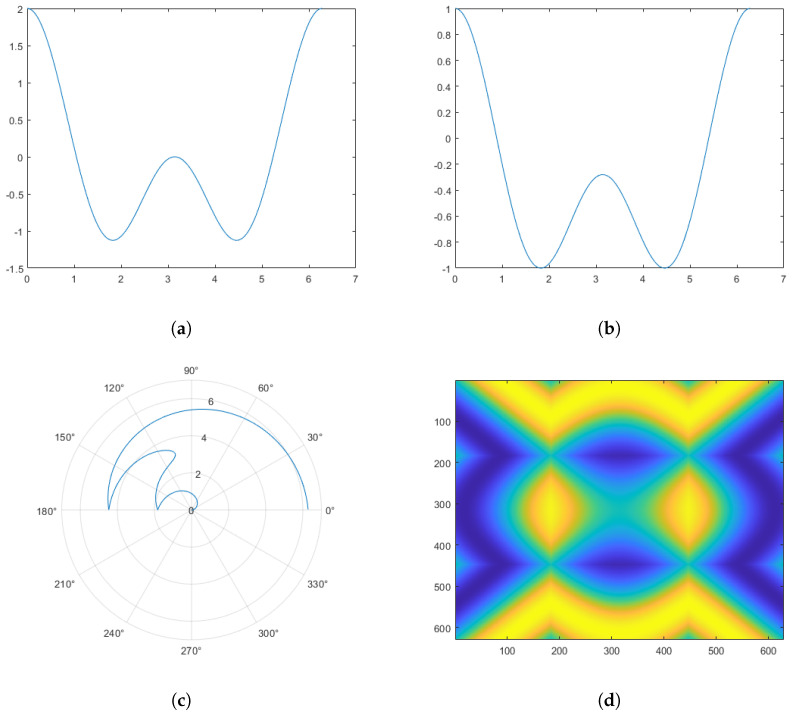
Illustration of GADF computation. (**a**) Original signal. (**b**) Normalized signal. (**c**) Mapping normalized signal to polar coordinates. (**d**) GADF.

Figure 2 provides a visual summary on the GADF computation of a one-dimensional signal.

A dedicated classification model is trained independently for each channel (i∈{1,2,…,N}) using the GADF representation. These models learn channel-specific discriminative features corresponding to different human actions. Pre-trained CNNs were fine-tuned as classifiers to leverage their learned feature representations while adapting them to the specific task of WiFi CSI-based human action recognition. The fine-tuning process began by initializing the network with weights from a CNN pre-trained on a large-scale dataset, such as ImageNet [30], which captures general visual features. The final fully connected layer of the pre-trained network, designed for the original classification task, was replaced with a new randomly initialized layer tailored to the number of classes in the target dataset. The network was then trained end-to-end using the GADF representations of the CSI data as input, employing a single learning rate for all layers to allow for simultaneous adaptation of both the pre-trained and newly added layers. This approach allowed the pre-trained CNN to serve as a feature extractor while fine-tuning it to achieve high classification accuracy on the task-specific data. To identify the most suitable architecture for the task, we evaluated the performance of seven different pre-trained CNNs (MobileNetV2 [49], ResNet18 [50], ResNet50 [50], ResNet101 [50], VGG16 [51], DenseNet201 [52], GoogLeNet [53]), fine-tuning each on the target dataset and comparing their classification accuracy. The parameters used for fine-tuning are summarized in Table 1. The cross-entropy loss function was utilized for optimization, and the Adam optimizer [54] was employed with parameters $\beta_{1}=0.9$, $\beta_{2}=0.99$, and $\epsilon_{2}=1\cdot 10^{-9}$. The learning rate was set to 0.0003, with a decay rate of 0.8 applied. The batch size was fixed at 32, and the model was trained for 10 epochs. The experiments were conducted using a computer with the following configuration summarized in Table 2. The system was built on a STRIX Z270H Gaming motherboard and operated on Windows. It featured an Intel(R) Core(TM) i7-7700K CPU running at 4.20 GHz with 8 cores. The system was equipped with 15 GB of RAM and utilized an Nvidia GeForce GTX 1080 GPU [55] for computational tasks. The implementation of the proposed method was carried out using Python version 3.11.5 and the PyTorch framework version 2.5.1. Python served as the primary programming language for developing and executing the code, while PyTorch provided the deep learning tools and libraries necessary for model training and evaluation.

A new WiFi signal is collected during the testing phase to evaluate system performance. Similar to the training phase, CSI data are extracted for each channel from the test signal. Each channel’s CSI is transformed into its corresponding GADF representation. The trained channel-specific models generate predictions based on the input GADF. The predictions from all channel-specific models are aggregated using a decision fusion mechanism to produce a final action classification result. This step combines channel-specific insights to enhance recognition accuracy and system robustness. The decision fusion mechanism employed in the system is based on majority voting, where the final classification result is determined by aggregating the predictions from all channel-specific models. Each model casts a “vote” for a particular class based on its prediction, and the class receiving the majority of votes across all models is selected as the final output. This approach enhances robustness by leveraging the collective decisions of multiple independently trained models, reducing the impact of errors from any single model.

## 5. Results

This section presents the experimental results and analysis of the proposed method. First, the evaluation metrics and protocol used to assess model performance are described. Next, the numerical results are provided, highlighting the effectiveness of the proposed approach compared to baseline methods and alternative architectures.

### 5.1. Evaluation Metrics and Protocol

Accuracy is a widely used evaluation metric for classification problems, particularly when the dataset is balanced [56]. For a multi-class classification task, accuracy is defined as the proportion of correctly predicted instances to the total number of instances in the dataset. Mathematically, it can be expressed as follows:

(6) $$\mathrm{Accuracy}=\frac{\mathrm{Number}\;\mathrm{of}\;\mathrm{correct}\;\mathrm{predictions}}{\mathrm{Total}\;\mathrm{number}\;\mathrm{of}\;\mathrm{predictions}}=\frac{\sum_{i=1}^{N}I\left(y_{i}={\hat{y}}_{i}\right)}{N},$$

where *N* is the total number of samples, $y_{i}$ is the true label for the *i*th instance, ${\hat{y}}_{i}$ is the predicted label for the *i*th instance, and I(·) is the indicator function that evaluates to 1 if its argument is true (i.e., $y_{i}={\hat{y}}_{i}$) and 0 otherwise.

**Figure 3:**
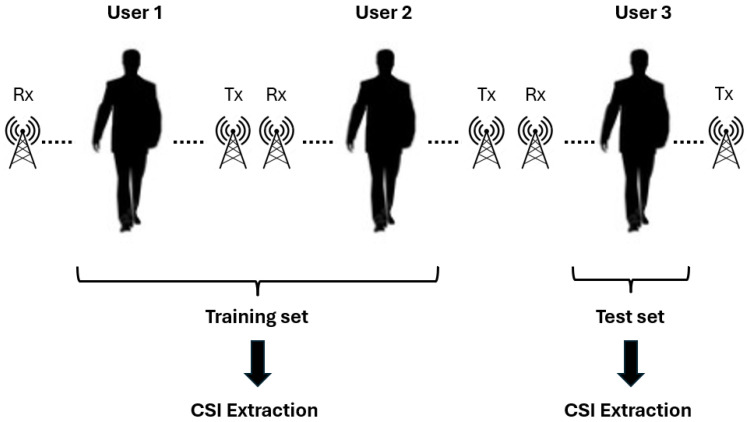
The evaluation procedure for a WiFi CSI-based human action recognition system on CSI-HAR dataset [40]. Data collected from Users 1 and 2 form the training set, while data from User 3 are used for testing. Channel state information is extracted from the received WiFi signals for both training and testing phases. This setup ensures evaluation of the model’s ability to recognize actions of new, unseen individuals.

The evaluation protocol is depicted in Figure 3. The evaluation protocol utilizes a user-specific train-test split to assess the performance of the human action recognition system. Namely, the CSI-HAR dataset [40] consists of CSI data collected from multiple users (e.g., User 1, User 2, and User 3), where each user’s movements are recorded using WiFi transmitter (Tx) and receiver (Rx) antennas. The CSI data from User 1 and User 2 are used as the training set. These data are extracted and processed to capture channel state information corresponding to the movements of these users. The CSI data from User 3 are reserved as the test set. This ensures that the evaluation is performed on an entirely unseen user, simulating a realistic scenario where the system generalizes to new individuals. For both the training and test sets, CSI is extracted from the raw WiFi signals to generate channel-specific data. This information is then used for feature extraction and classification during the training and testing phases. This leave-one-user-out approach ensures that there is no overlap between the training and testing data, providing a robust evaluation of the model’s ability to generalize to new users. The evaluation protocol on the Basic Knife Skills Database [43] is similar to those of CSI-HAR [40]. However, unlike CSI-HAR [40] with three humans, this one includes only two. Consequently, the data from the first human are used for the training set, while the data from the second human are allocated to the test set.

### 5.2. Numerical Results

**Figure 4:**
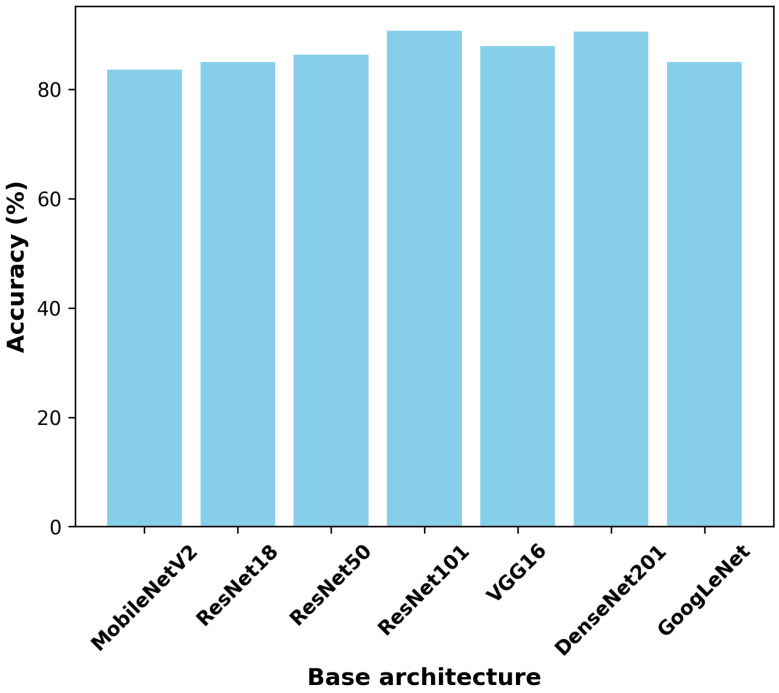
Accuracies with respect on different base architectures, measured on CSI-HAR dataset [40].

**Figure 5:**
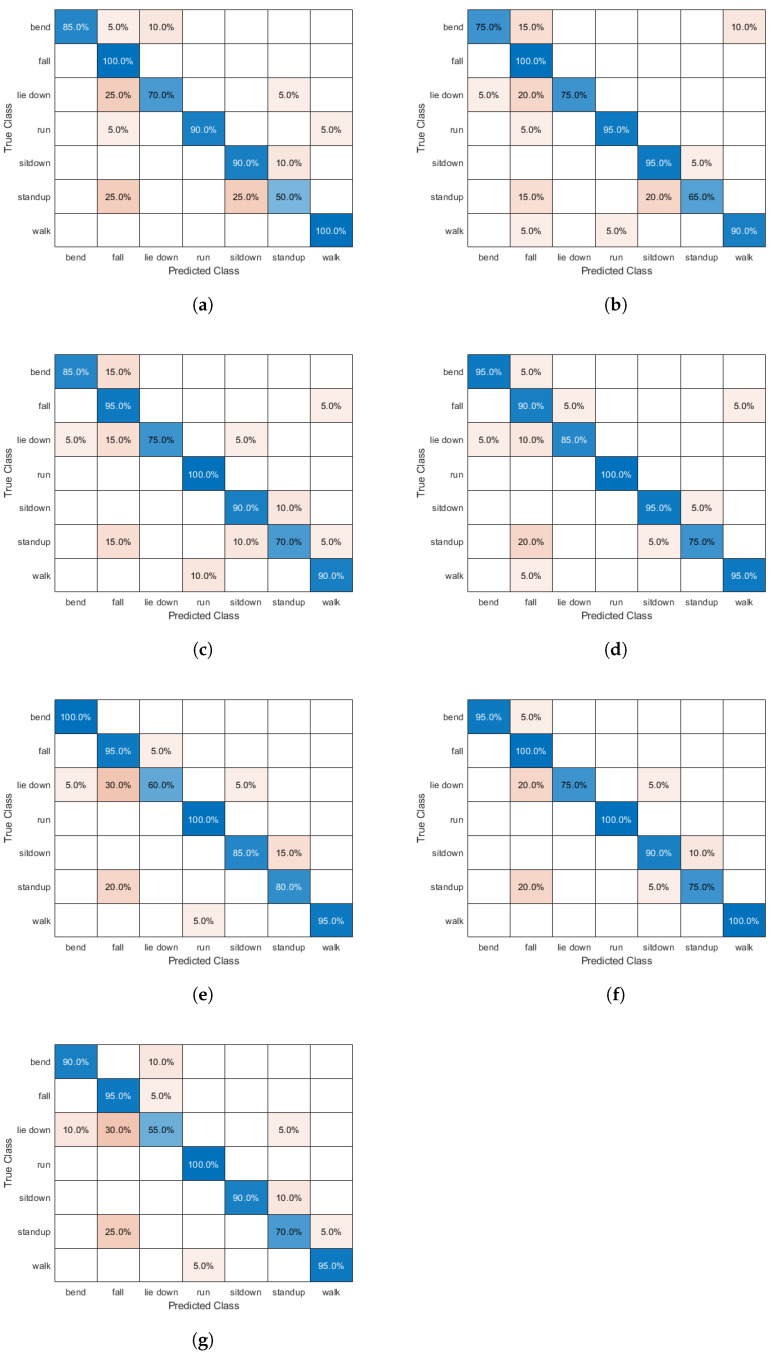
Confusion matrices with respect to different base architectures. (**a**) MobileNetV2. (**b**) ResNet18. (**c**) ResNet50. (**d**) ResNet101. (**e**) VGG16. (**f**) DenseNet201. (**g**) GoogLeNet.

Figure 4 illustrates the comparison of various pre-trained CNN architectures as potential base models for the proposed method. The architectures evaluated include MobileNetV2 [49], ResNet18 [50], ResNet50 [50], ResNet101 [50], VGG16 [51], DenseNet201 [52], and GoogLeNet [53]. Among these, ResNet101 [50] achieved the highest accuracy, demonstrating its superior performance for the task. Other architectures, such as DenseNet201 [52] and VGG16 [51], also performed well but fell slightly short of ResNet101 [50]. Models like MobileNetV2 [49] and GoogLeNet [53] exhibited lower performance in comparison. Based on these results, ResNet101 [50] was selected as the base architecture for its optimal depth and feature extraction capabilities. The resulting structure, codenamed DF-CNN (where “DF” refers to decision fusion), integrates the strengths of ResNet101 [50] to achieve robust human action recognition using WiFi CSI data. The confusion matrices of the different base architectures can be seen in Figure 5, providing further insight into their classification performance. The confusion matrices display true classes on the y-axis and predicted classes on the x-axis, with color intensities representing classification frequencies. The considered activity classes include bend, fall, lie down, sit down, stand up, standing, and walk. Diagonal elements represent correctly classified instances, whereas off-diagonal elements indicate misclassifications. Across all architectures, standing, falling, and running are generally well-classified, showing high accuracy. However, fall, lie down, and sit down appear to be more challenging for most models, with frequent misclassifications among them. In particular, fall is often confused with lie down, while sit down is sometimes misclassified as stand up or standing. The comparison highlights variations in model performance, offering insights into robustness and misclassification tendencies. These findings are valuable for selecting the most effective architecture for human activity recognition, where certain misclassifications (e.g., failing to detect a fall) could have critical implications.

**Figure 6:**
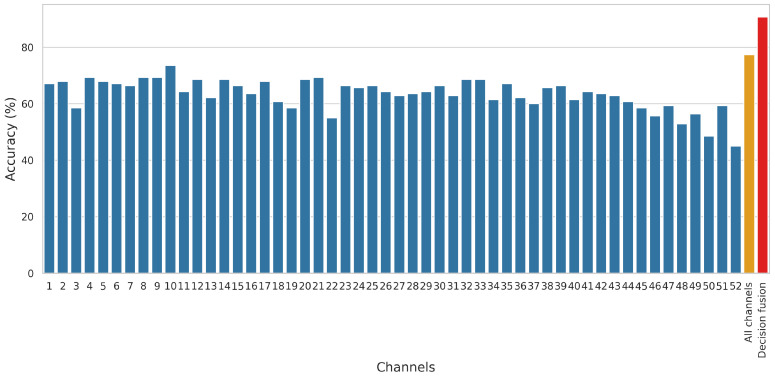
Parameter study examining the performance of ResNet101 [50] on individual CSI channels, all channels combined, and the proposed DF approach. Each bar represents the accuracy achieved by treating the channels separately or together. In the combined approach, all channels were included in the training and test sets as if they represented a single channel. It can be observed that the decision fusion method significantly outperforms both individual channel evaluation and the combined use of all channels, demonstrating its effectiveness in leveraging channel information.

To analyze the impact of different CSI channels on the model’s performance, we conducted a parameter study where the channels were evaluated individually, as well as in a combined manner. In the combined approach, all channels were treated as a single input, meaning that both the training and test sets consisted of data from all channels as if they represented a single channel. The results, depicted in Figure 6, reveal that the combined approach improves accuracy compared to evaluating individual channels. However, the proposed DF approach outperforms both strategies, achieving the highest accuracy. This demonstrates that DF effectively leverages information across channels, providing a more robust and accurate solution for human action recognition.

Table 3 presents a comparative analysis of various WiFi CSI-based human action recognition methods evaluated on the CSI-HAR dataset. The examined state-of-the-art methods were also implemented using Python 3.11.5 and PyTorch 2.5.1 to ensure consistency in the experimental setup. By utilizing the same tools and frameworks, any performance differences could be attributed solely to the models rather than implementation discrepancies. Furthermore, the same evaluation protocol was applied across all methods—already given in Section 5.1—to ensure a fair and reliable comparison of their performance. Accuracy rates vary significantly across the methods. Traditional deep learning architectures like 2D-CNN [40] and 1D-CNN [40] achieved accuracies of 66.4% and 55.0%, respectively. Recurrent models, such as LSTM [40] and BLSTM [40], realized accuracies of 61.8% and 62.2%. Different CNN-based approaches, including CNN-Plain [57], CNN-Canny [57], CNN-Sobel [57], CNN-Prewitt [57], and CNN-LoG [57], attained accuracies between 58.6% and 61.4%. More advanced models, such as Jawad et al. [58] and ImgFi [29], demonstrated improved performance with accuracies of 77.1% and 79.1%, respectively. The DF-CNN (proposed) method significantly outperformed all other approaches, achieving an accuracy of 90.7%, highlighting its superior ability to classify human actions effectively. This analysis underscores the advancements achieved by the proposed DF-CNN model in addressing the challenges of human action recognition using WiFi CSI data. Table 4 presents a comparison of results on the Basic Knife Skills Database [43]. It can be seen that accuracy values range from 23.33% to 33.3%. The low accuracy values observed across the methods can be attributed to the limited number of samples available in this database. Additionally, the database consists of data collected from only two individuals, which further restricts its variability and the ability of models to generalize effectively. Notably, the proposed method (DF-CNN) achieves the highest accuracy at 33.3%, outperforming all other methods listed. Methods such as CNN-Sobel [57], CNN-Prewitt [57], and CNN-LoG [57] show slightly higher accuracy values (30.00%) compared to other methods, while 1D-CNN [40], LSTM [40], and ImgFi [29] demonstrate lower accuracy (23.33% and 26.67%, respectively).

**Figure 7:**
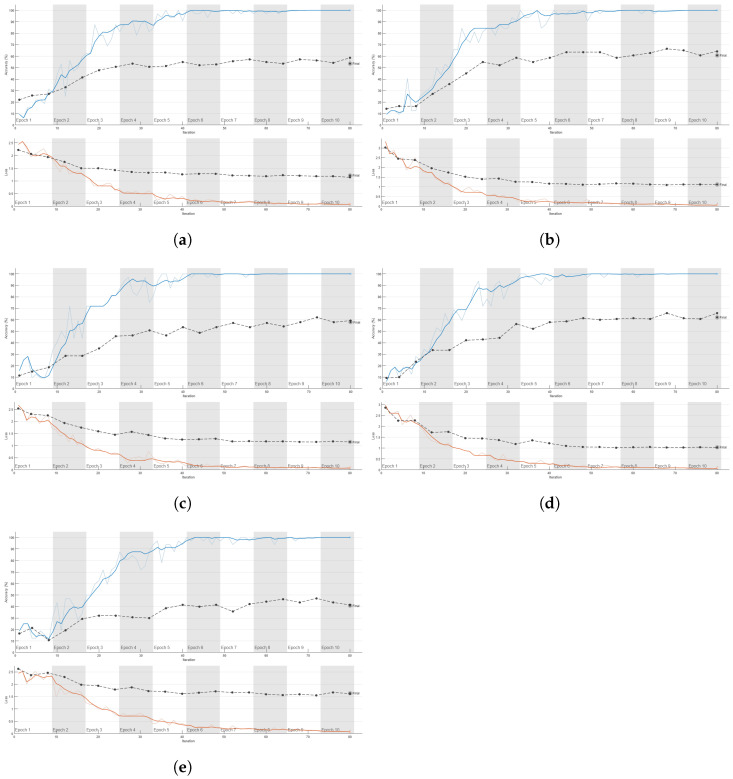
Training curves of ResNet101 on different CSI channels. Each subfigure represents the performance metrics (e.g., accuracy and loss) across epochs for a specific CSI channel, illustrating the model’s training dynamics and convergence behavior. Dashed lines indicate performance metrics (accuracy and loss) on the test set. (**a**) 1st CSI channel. (**b**) 13th CSI channel. (**c**) 26th channel. (**d**) 39th channel. (**e**) 52th channel.

Figure 7 depicts the training curves of ResNet101 [50] architecture of five different CSI channels using the CSI-HAR database [40]. Specifically, the image presents a set of training curves for ResNet101 on different CSI channels, illustrating the model’s training dynamics and convergence behavior. Each subfigure (a–e) corresponds to a specific CSI channel: 1st, 13th, 26th, 39th, and 52nd channels, respectively. The curves plot accuracy (blue) and loss (red) across training epochs, with dashed lines indicating performance on the test set. Across all CSI channels, the accuracy generally increases over epochs, while the loss decreases, demonstrating effective training convergence. However, variations exist in the rate and stability of convergence across different channels. Some CSI channels exhibit more stable and rapid convergence, while others show more fluctuations, suggesting potential differences in their discriminative power for classification tasks.

## 6. Discussion

This study introduced DF-CNN, a novel architecture that processes WiFi CSI channels separately and leverages decision fusion to enhance human action recognition. The results clearly demonstrate the advantages of this approach over traditional methods, which treat all CSI channels as a single input. By evaluating individual channels and employing a fusion mechanism, DF-CNN achieved a significant performance improvement, setting a new standard in the field.

A key finding of this work is the superior performance of DF-CNN compared to both individual channel evaluation and the combined approach, where all channels are treated as one. The combined approach, while effective to some extent, fails to fully capture the distinct characteristics of each channel. In contrast, the decision fusion strategy harnesses these unique features, allowing DF-CNN to achieve state-of-the-art accuracy. This highlights the importance of channel-specific processing in leveraging the full potential of CSI data.

Another critical observation is the effectiveness of ResNet101 as the base architecture for DF-CNN. Through rigorous experimentation, ResNet101 consistently outperformed other pretrained models, demonstrating its suitability for capturing complex spatial and temporal patterns in CSI data. This suggests that the choice of base architecture is pivotal for achieving optimal performance in CSI-based human action recognition.

The proposed DF-CNN not only advances the field technically but also addresses key limitations of prior works. Many existing methods report inflated performance metrics due to data leakage, which arises from improper data partitioning strategies. Our approach mitigates this issue by adopting a robust evaluation protocol, ensuring the reported results are both reliable and reproducible [59]. Despite its success, DF-CNN also opens avenues for future research. While the decision fusion approach proved highly effective, alternative fusion strategies, such as attention mechanisms or ensemble learning, could further enhance performance. Additionally, the scalability of DF-CNN to larger datasets and more diverse action recognition scenarios warrants further investigation [60].

In conclusion, the proposed DF-CNN architecture introduces a paradigm shift in CSI-based human action recognition by emphasizing channel-specific processing and decision fusion. This work underscores the importance of treating CSI data with careful consideration of its inherent structure and paves the way for future advancements in non-invasive human activity recognition systems.

## 7. Conclusions

In this study, we presented DF-CNN, a novel architecture for human action recognition using WiFi CSI. Unlike traditional methods that treat all CSI channels collectively, our approach processes each channel separately and integrates their outputs using a decision fusion strategy. This design capitalizes on the unique characteristics of individual channels, resulting in a significant performance improvement over existing techniques. Extensive experiments demonstrated the effectiveness of DF-CNN, with the decision fusion strategy outperforming both individual channel evaluation and the combined approach. The use of ResNet101 as the base architecture further contributed to the model’s superior performance, showcasing its ability to capture the complex patterns inherent in CSI data. These findings establish DF-CNN as a robust and reliable framework for CSI-based human action recognition. In summary, DF-CNN offers a new perspective on CSI data processing by demonstrating the importance of channel-specific treatment and decision fusion. This contribution advances the state of the art in non-invasive human action recognition, which could be applied in home activity monitoring, healthcare monitoring, smart energy management, or occupancy detection, and sets a benchmark for future developments in the field. 

## Figures and Tables

**Table 1:** Parameter setting.

Parameter	Value
Loss function	Cross-entropy
Optimizer	Adam [54] $\left(\beta_{1}=0.9,\beta_{2}=0.99,\epsilon_{2}=1\times 10^{-9}\right)$
Learning rate	0.0003
Decay rate	0.8
Batch size	32
Epochs	10

**Table 2:** Computer configuration.

Computer model	STRIX Z270H Gaming
Operating system	Windows
CPU	Intel(R) Core(TM) i7-7700K CPU 4.20 GHz (8 cores)
Memory	15 GB
GPU	Nvidia GeForce GTX 1080

**Table 3:** Comparison of results on CSI-HAR [40].

Method	Accuracy
2D-CNN [40]	66.4%
1D-CNN [40]	55.0%
LSTM [40]	61.8%
BLSTM [40]	62.2%
CNN-Plain [57]	58.6%
CNN-Canny [57]	61.4%
CNN-Sobel [57]	60.4%
CNN-Prewitt [57]	59.7%
CNN-LoG [57]	61.0%
Jawad et al. [58]	77.1%
CNN [28]	77.5%
ImgFi [29]	79.1%
DF-CNN (proposed)	**90.7%**

**Table 4:** Comparison of results on Basic Knife Skills Database [43].

Method	Accuracy
2D-CNN [40]	26.67%
1D-CNN [40]	23.33%
LSTM [40]	23.33%
BLSTM [40]	23.33%
CNN-Plain [57]	26.67%
CNN-Canny [57]	28.33%
CNN-Sobel [57]	30.00%
CNN-Prewitt [57]	30.00%
CNN-LoG [57]	28.33%
Jawad et al. [58]	28.33%
CNN [28]	26.67%
ImgFi [29]	26.67%
DF-CNN (proposed)	**33.3%**

## Data Availability

The data used in this study were derived from the CSI-HAR database available for download at https://github.com/parisafm/CSI-HAR-Dataset (accessed on 19 December 2024). For this study, the data were rearranged to address specific research questions, and the rearranged dataset is available for download at https://github.com/elektrische-schafen/CSI-HAR-Database (accessed on 19 December 2024). The Basic Knife Skills Database is available at https://ieee-dataport.org/documents/wifi-csi-and-rssi-data-six-basic-knife-activities-cooking-chopping-cubing-french-cutting (accessed on 19 December 2024).

## Abbreviations

The following abbreviations are used in this manuscript:

BLSTM	bi-directional long short-term memory
CNN	convolutional neural network
CSI	channel state information
DF	decision fusion
GAF	Gramian angular field
GADF	Gramian angular difference field
IEEE	Insitute of Electrical and Electronics Engineers
LoG	Laplacian of Gaussian
LSTM	long short-term memory
NISE	N-iteration signal enhancement
OFDM	orthogonal frequency division multiplexing
PSE	P-signal enhancement
RNN	recurrent neural network
RSSI	received signal strength indicator
SVM	support vector machine
